# Language barriers and their consequences in healthcare: a qualitative case study of Nepali migrants in Finland

**DOI:** 10.1186/s12913-025-12757-y

**Published:** 2025-04-22

**Authors:** Shrwan Kumar Khanal

**Affiliations:** Faculty of Social Sciences, University of Helsinki, Helsinki, Finland

**Keywords:** Migrants’ health, Language barriers, Informal networks, Nepali migrants, Interpreter services, Healthcare rights, Co-ethnic networks, Language concordance care, Occupational Healthcare, Minority Migrants

## Abstract

**Background:**

Migrants face multiple barriers in using healthcare, with language being a major obstacle. In Finland, research on minority migrant groups, such as Nepali migrants, remains limited. This study examines how language barriers, informal networks, and interpreter services affect healthcare use among the Nepali migrant community, a small but growing group in Finland.

**Methods:**

This qualitative study employed semi-structured interviews with 27 working age Nepali migrants in Finland. The data was collected between February and August 2023 and was analysed thematically to identify barriers to healthcare use.

**Results:**

Limited language proficiency restricted participants’ ability to navigate healthcare services, comprehend medical instructions, and communicate effectively with providers, which in some cases contributed to experiences of perceived discrimination. Consequently, many relied on informal networks, such as employers or co-ethnic communities, for healthcare information, often receiving misleading or incomplete guidance. The shortage of professional interpreters further worsened these challenges, while privacy concerns discouraged the use of community-based interpreters. Moreover, participants’ limited awareness of their healthcare rights as employees reinforced their dependence and increased their vulnerability to labour exploitation within migrant communities.

**Conclusions:**

Language barriers not only affect direct communication but also contribute to systemic inequalities in healthcare access by reinforcing reliance on informal support structures. Addressing these challenges requires providing language training programs, enhancing the availability of professional interpreters, and ensuring that new migrants are informed about their healthcare entitlements through effective integration programs tailored to minority migrants.

## Background

Healthcare access for minority populations, particularly migrants, is a critical public health issue in many migrant-receiving countries. While access to healthcare is a fundamental right, many migrants face significant structural, linguistic, and informational barriers that impede their ability to navigate the system, leading to unmet health needs and disparities in health outcomes [1, 2].

For migrants in Europe, access to healthcare is often hindered by a combination of factors. Distinct national languages, along with the communication challenges and potential discrimination migrants may face, all contribute to inadequate healthcare access [3–5]. Despite legal entitlements to equal healthcare access in Nordic countries, disparities persist between migrants and non-migrants [6–8]. These disparities have been attributed to limited knowledge of the healthcare system, information predominantly provided in the local language, and a lack of cultural competency among providers. Also, the shortage of trained interpreters and reliance on family members or ad hoc community interpreters has been linked to adverse health outcomes among migrants in Nordic countries [9, 10] such as Finland [11], which is the focus of this paper.

### Migrants in Finland

Migration to Finland is a relatively recent phenomenon. The number of migrants in Finland has steadily increased, rising from less than 1% of the population in 1990 to 10.2% of the country’s 5.6 million residents in 2023 [12]. Initially, migration to Finland was primarily driven by humanitarian concerns. However, in recent years, the country has increasingly welcomed economic and skilled migrants to address its ageing population and support its high-tech industries. This growing diversity is reflected in Finland’s evolving linguistic landscape. Although Finnish, Swedish, and Sami remain the national languages, spoken by nearly 90% of the population, a wider range of languages is now in use. Beyond the national languages, Russian and Estonian are the most commonly spoken foreign languages, alongside a growing presence of Arabic, English, Kurdish, Somali, and Persian.

In 2022, 58% of Finland’s foreign-language-speaking population resided in the Helsinki capital region. This concentration is projected to increase, and the City of Helsinki anticipates a growing presence of Asian languages, particularly Chinese, Vietnamese, and Nepali, by 2030 [13].

### Nepali migrants in Finland

Nepali migrants represent a small but rapidly expanding segment of Finland’s migrant population, experiencing considerable growth since 2015. By 2023, over 7,000 Nepali migrants resided in Finland, with a notable concentration (70%) in the capital region. This trend has continued, with nearly 3,000 new residence permits issued to Nepali nationals between February 2024 and January 2025, making them the third-largest group among new arrivals after migrants from India and the Philippines [14].

A significant majority (82%) of Nepali migrants in Finland are of working age (15–74 years), with a substantial proportion (73%) aged between 20 and 45 [12]. Employment, education, and family reunification are the primary drivers of this migration. Nepali restaurants, estimated unofficially to number over 150 and predominantly concentrated in the capital region, serve as key employers and provide a visible example of ethnic entrepreneurship within this relatively small population. Recent media attention on Nepali restaurants in Finland has highlighted serious concerns, including legal cases and investigations related to human trafficking, labour violations, and the tragic suicide of a Nepali worker at his workplace [15–17]. These incidents highlight critical issues regarding the well-being and healthcare access of Nepali workers in Finland. A discussion of the Finnish healthcare system and its integration policies is crucial for understanding the context in which these experiences occur.

### Healthcare provision in Finland

In Finland, legislation guarantees comprehensive health and social services to all individuals, regardless of their resident status. These services include public healthcare, Occupational Healthcare (OHC) mandated by employers, and private healthcare [18]. Primary healthcare is administered by wellbeing service counties and delivered through health centres. Specialised healthcare, which requires a referral and is primarily provided in hospitals, offers more targeted examinations and treatments [19]. Employers are required to provide preventive healthcare under Occupational Safety and Health regulations, with some also offering additional medical care as an employee benefit. OHC services are often contracted through private companies.

Private healthcare provides a supplementary option to the public system, with costs typically covered by individuals directly or through private health insurance. However, research suggests that migrants in Finland use OHC and private healthcare less frequently compared to the general population [20, 21]. Furthermore, Finland is a leader in digital infrastructure, and digital technologies and online platforms are widely used to deliver public services, including healthcare. While a significant portion of health and social service information is available digitally, it is primarily in Finnish and Swedish, with limited availability in English.

### Integration policies of Finland

Recognising the rapid growth in migration and the associated challenges of integration in Finland, the Finnish government offers integration programs tailored to specific migrant groups. These programs, primarily designed to facilitate rapid entry into the job market, include language training, job search assistance, on-the-job training, and opportunities for further education [22]. These initiatives are available for the first three years following a migrant’s arrival but typically exclude those who migrate to Finland specifically for employment or study purposes [23]. Beyond these formal programs, migrants can also pursue language learning through various avenues, including adult education centres, workers’ institutes, folk high schools, universities, and summer universities.

The Finnish Language Act (2003/423) [24] guarantees access to interpreters for individuals who do not understand the official languages when using public services, including healthcare. Despite this, research suggests that interpreter services’ availability, reliability, and effectiveness remain problematic [11, 25].

These practical challenges are particularly pronounced for smaller migrant communities, where structural support might be less accessible or consistent despite national policies and legal provisions aiming to ensure equitable access to services.

### Exiting research in Finland

Inclusive healthcare access for Finland’s growing migrant population remains a challenge. Earlier research has identified barriers such as high costs, communication difficulties, long waiting times, limited knowledge of the healthcare system, and negative experiences with providers [26]. Additionally, culturally insensitive services, differing perceptions of illness, stigma, and limited language proficiency contribute to unmet healthcare needs, leading to lower satisfaction and reduced access compared to the general population [20, 27, 28]. Past traumatic experiences, social networks, and trust in healthcare services further influence healthcare use. Migrants from the Middle East and North Africa, in particular, experience high levels of psychological distress associated with residency status, economic inactivity, and language barriers [29, 30].

Social and economic integration challenges further worsen these difficulties. A recent study reports that language barriers, employment discrimination, racism, income inequality, and limited integration opportunities hinder migrants’ access to healthcare in Finland [31]. Moreover, the fragmented structure of Finnish social services complicates access by making it difficult to communicate with service providers, locate necessary services, and establish contact with healthcare professionals [18, 32]. Together, these perspectives help to explain why access to public services including healthcare services remains uneven among migrant populations.

### Research gap and study objectives

The challenge of language barriers in healthcare access is well-documented. Despite this, research on how these barriers specifically affect individual migrant communities, for instance, Nepalis in Finland, is limited. Current research tends to focus on larger migrant groups from neighbouring countries or humanitarian arrivals, leaving the experiences of economic migrants relatively unexplored, despite their potentially unique healthcare challenges. This study addresses this gap by examining how language barriers influence Nepali migrants’ access to healthcare in Finland. Specifically, this research investigates:

- How language barriers influence Nepali migrants’ ability to navigate the Finnish healthcare system, access medical services, and understand their healthcare rights.
- The role of informal networks (including co-ethnic communities and employers) in shaping healthcare decisions and the potential spread of misinformation.
- Challenges related to interpreter services encompassing availability, quality, privacy concerns, and their overall impact on healthcare experiences.

By focusing on these key areas, this study will provide valuable insights for policymakers seeking to improve healthcare accessibility and workplace protection for migrants. Furthermore, by focusing on an economic migrant group with potentially distinct cultural healthcare practices, the research will challenge the universality of standardised healthcare policies and underscore the need for more adaptable and inclusive approaches to migrant healthcare.

## Methods

### Research design

This study employed a qualitative research design, using semi-structured interviews [33] to explore the experiences of working age Nepali migrants residing in the capital region of Finland. The research aimed to capture in-depth insights from participants across a diverse range of socio-demographic backgrounds. In this study, “migrants” refers to individuals of foreign origin who have relocated to Finland, including those who have acquired Finnish citizenship through naturalisation.

### Recruitment of participants

Information about the study was initiated by posting in widely used Nepali Facebook groups in Finland, inviting volunteer participation. Interested individuals contacted the researcher and were screened based on background information. A purposeful sampling technique [34] was employed to select participants, ensuring the representation of various characteristics. This approach combines purposive, convenience, and snowball sampling methods to identify suitable participants. The inclusion criteria required participants to have lived in Finland for at least one year, be within the working-age group (15 to 74 years) and have some experience with the Finnish healthcare system. The recruitment process further ensured diversity in age, sex, length of stay, educational background, Finnish language proficiency, and digital literacy.

Given the occupational distribution of Nepali migrants, participants were purposively selected, including employees and employers from Nepali restaurants, as well as health and social care workers. The researcher’s prior experience in the food industry, health and social care services, and community activism facilitated access to suitable participants, resulting in some familiar individuals being included in the study. Additionally, some participants were approached and selected, as interviewees referred others, they believed would contribute valuable insights. In total, 31 participants were approached, but four individuals who did not meet the inclusion criteria were excluded.

### Data collection

All participants were initially contacted by phone, during which the study’s objectives and their roles were explained. Before the interview started, all participants received a consent form and an information letter in Nepali, outlining the study’s purpose, ensuring confidentiality, and informing them of their right to withdraw at any time without providing a reason. The study protocol was reviewed and approved by the Research Ethics Committee in the Humanities and Social and Behavioural Sciences at the University of Helsinki (12/2023).

Data were collected through 27 face-to-face interviews conducted between February and August 2023. The interviews were carried out by the author, a member of the same migrant community. To ensure participants could express themselves comfortably, all interviews were conducted in Nepali, their native language. An interview guide [35] was developed based on existing literature on migrant healthcare experiences to structure the conversation. While the guide provided a structured framework, it remained iterative, allowing for new themes and follow-up questions to emerge as the interview transcripts were thoroughly read.

A few participants were identified via the researcher’s network before the data collection process started. The first interview was conducted with an entrepreneur. Subsequent interviews included employees and health and social care professionals. Additionally, recommended participants identified through the snowball sampling method, were approached and interviewed. Engaging with individuals from different professional backgrounds contributed to refining the interview questions. Interviews took place in a variety of settings: seven were conducted in a library, six at the researcher’s residence, and fourteen at the participants’ homes. While interview durations varied from 19 to 61 min, the average length was 42 min. The shortest interview was due to a miscommunication concerning the library’s booking system but included important information on the topic.

### Data analysis

The data was transcribed verbatim, and inductive thematic analysis was used, following the six-phase process outlined by Braun and Clarke [36]. The analysis began with familiarisation with the data through repeated reading of the interview transcripts. Initial codes were generated based on both the existing literature and the interview transcripts. These codes were then clustered into potential themes, which were further developed and reviewed to ensure coherence and relevance. The analysis continued with mapping patterns and exploring relationships between the themes, ultimately leading to the identification of generalised descriptions by merging themes and subthemes.

Throughout the analysis, the researcher was aware of the potential bias that could arise because both the researcher and participants were part of the same migrant groups, a situation known as “insider status” [37]. Some participants viewed the researcher as an assistant, which made them more open to discussing sensitive topics such as employment discrimination and challenges related to social integration. For instance, some asked, “Can you help me get a B-Statement (A doctor’s certificate that would allow relatively long sick leave)?” While this insider position proved valuable to the study, there were instances where participants were more hesitant, possibly perceiving the “fear of negative repercussions” [37]​​ in their job-related contexts. For example, some asked, “Why should I tell you this? Won’t it affect me?” A research diary [38] was maintained to document observations, reflections, and the potential influence of insider status. The diary also recorded instances where participants recalled key information after the recording stopped or chose to keep certain details unrecorded. These notes were considered during the data analysis.

The study’s credibility was strengthened by implementing several established methodological strategies (see, for example, Greene [39]) to minimise potential biases, including insider bias. Specifically, reflexivity was maintained through active self-reflection on positionality and biases. Triangulation was achieved by comparing findings with existing literature and engaging in discussions with peers and migration researchers. Transferability was reinforced by meticulously documenting analytical decisions in a research diary. Finally, peer debriefing with migration researchers was used to refine interpretations and deepen the analysis.

### Participants’ characteristics

The study included 27 participants: 13 men and 14 women. Their ages ranged from 31 to 67 years, with an average age of 44. The participants had lived in Finland for 4 to 29 years, with an average residence duration of 12 years. Of the participants, five were practical nurses employed in health centres, hospitals, and care homes. In addition, nine participants represented the restaurant industry, comprising five employers and four chefs. The other thirteen participants worked in other service sectors, such as delivery and warehousing, or were currently unemployed. Other characteristics of the participants are shown in Table 1.

**Table 1:** Participants’ characteristics

Characteristics	*N*
Gender	
Man	13
Woman	14
Highest level of education*	
Basic (up to 10 years)	9
Higher secondary (11–12 years)	5
University level (13 + years)	13
Age	
30–34	6
35–39	5
40–44	5
45–49	6
50+	5
Profession	
Entrepreneur	4
Restaurant chef	5
Health and social care sector	5
Other service sector	9
Others	4
Self-assessed Finnish skills	
Poor	13
Medium or high	14
Self-assessed digital Skills	
Poor	12
Medium	9
High	6
Number of years in Finland	
1–9	10
10–14	9
15–19	4
20+	4
Reason for migration	
Work	9
Family reunification	12
Study	6

*This refers to the education completed in Nepal

## Results

This section presents the barriers faced by Nepali migrants in accessing and using healthcare services in Finland. The findings are organised into three themes: language barriers and healthcare challenges; informal networks as a source of health information; and interpreter services and privacy problems.

### Language barriers and healthcare challenges

Language barriers had a considerable impact on participants’ ability to access healthcare services. A lack of proficiency in the local language was reported as a barrier in various processes, such as understanding medication instructions in the healthcare system and communicating with healthcare providers. Although healthcare information was available, language barriers rendered it inaccessible.

Additionally, differences between the healthcare systems in Nepal and Finland further complicated migrants’ understanding of procedures. Unlike Nepal, where individuals can go directly to any hospital, in Finland, clients must visit a health centre first and obtain a referral. This difference caused frustration as noted:

“When I wanted to go to the hospital, they kept saying ‘Terveysasema, Terveysasema’ (health centre in Finnish), and we almost quarrelled. It was so frustrating because we don’t have this system in Nepal. I didn’t understand the difference between a health centre and a hospital or where I should go.” (Participant 7, Man)

Students also reported similar difficulties in navigating the services offered by different health service providers:

“I didn’t know whether to go to student healthcare or a local health centre. I had insurance from Nepal, but it only covered emergencies. When I called the local centre, they said I should see a student health doctor. I didn’t get proper guidance. Now I’ve changed my residence status to employment, so I know how things work.” (Participant 21, Woman)

Participants also reported challenges in understanding prescriptions and instructions:

“When I got instructions for medication, it was all in Finnish. I had to rely on Google Translate and friends, but I wasn’t sure if I understood it right.” (Participant 17, Man)

“Once I gave the medicine to my kids orally, which was supposed to be inserted through the rectum. Imagine a highly educated person like me having this kind of experience. They might have told me, but maybe I didn’t understand properly.” (Participant 15, Woman)

“The doctor said something, but I didn’t fully understand. I took a 600 mg tablet as prescribed, but after a week, I felt depressed and couldn’t sleep. My son checked, and it turned out the medicine was very strong and meant to be split into three doses.” (Participant 19, Woman)

Language barriers also contributed to feelings of discrimination among the study participants. Those who tried communicating in English felt that healthcare staff responded differently, while others who struggled to describe symptoms in Finnish felt dismissed or ignored.

One participant highlighted the limitations of using English in public healthcare as:

“I can get appointments with English-speaking doctors through my work, but for my chronic illness, I need to go through the public health centre. When I speak in English, they don’t call me back for an appointment.” (Participant 9, Man)

Others shared similar concerns, noting that those fluent in Finnish received quicker appointments and better care. For example, one participant said:

“My coworkers who speak perfect Finnish get quick appointments, but I struggle to explain myself, and the staff deny me further appointments” (Participant 14, Woman)

Many participants stated that they could go “everywhere” for health check-ups if they understood the local language. They believed that individuals fluent in the local language could more effectively navigate different healthcare settings.

A significant number of participants stated that they did not have the opportunity to learn the language. Some did not feel the need to do so, as they were employed directly by Nepali employers upon their arrival, who also assisted in arranging their daily needs. However, they realised later this mistake:

“It’s our fault, we didn’t learn the language or technology [---].” (Participant 2, Man)

### Informal networks as a source of health information

Most Nepali migrants arriving in Finland utilise pre-existing networks, often finding employment as chefs in Nepali restaurants or joining family members already established in the country. Consequently, they heavily rely on these networks for all aspects of daily life, including navigating the healthcare system. Limited language skills led many migrants to rely on closed and informal healthcare networks.

A participant shared his experience in acquiring healthcare information:

“I got some leaflets about the healthcare system, but I didn’t pay attention because I don’t understand English or Finnish. What use is that information to me? I just followed what my boss said, but now I regret it.” (Participant 11, Man)

Participants reported that they received misleading information about their healthcare rights. One participant shared:

“My boss was very strict about going for check-ups. He said that foreigners don’t get free doctors until they have permanent residency. If I go for a minor illness, it costs a lot of money and also decreases my salary.” (Participant 11, Man)

Language barriers also complicate their efforts to familiarise themselves with the OHC and employees’ rights. For example, one participant explained how he only discovered his company’s healthcare coverage after he went to a health check-up:

“I had back problems and went to the hospital. They talked about facilities for workers, like safety shoes and sick leave. I found out later that my company should have covered some of these costs, but I had to rely on others for help. The doctors said treatment would be expensive, and after that, my employer did not allow me to go back to the hospital.” (Participant 2, Man)

These narratives suggest a potential link between migrants’ misunderstanding of their healthcare rights, including OHC, and their vulnerability to labour exploitation. Many participants expressed confusion about how accessing healthcare in Finland might affect their residency and employment status. For example, some feared that attending health check-ups before securing permanent residency could jeopardise their job security and ability to sponsor family reunification. This misconception was also present among individuals working in the social and healthcare sectors, who expressed uncertainty about the availability of OHC services for those in temporary positions (Participants 14, 19, and 26). These concerns likely contribute to the underutilisation of available healthcare services among this population.

### Interpreter services and privacy problems

Although language assistance is available through interpreters in Finland, participants struggled to access these services effectively. One recurring issue was the unavailability of interpreters, which frequently caused delays in receiving care. Participants expressed frustration over how these delays disrupted their treatment and wasted time. As one participant noted:

“I visited the doctor but due to the sudden cancellation of the tulkki (interpreter), I had to return home without treatment. So, I suggest tulkki (interpreter) not schedule time if they cannot manage it, why waste others’ time?” (Participant 12, Man)

In more severe cases, delayed access to interpreters resulted in postponed medical procedures, as noted by another participant:

“My case should have been treated earlier, but due to the unavailability of tulkki (interpreter), they postponed the operation. For those who lack the Finnish language, it is a problem everywhere.” (Participant 2, Man)

Participants were also concerned about the quality of translation. One participant noted the difficulty their interpreter faced in translating medical terms:

“It was monotonous to hear the conversation because the doctor spoke a few sentences, paused, and continued after the interpreter interpreted the speech. He was using Google Translate as well. The meaning was misunderstood as it reached me.” (Participant 22, Woman)

This indeed highlights how challenging it can be to convey messages accurately in healthcare, even for interpreters.

Similarly, another participant emphasised the risks of misinterpretation in critical medical situations:

“If some words are wrongly interpreted in the operation theatre, it may be life-threatening.” (Participant 20, Man)

These narratives highlight how inconsistent access to qualified interpreters negatively affects healthcare experiences, contributing to treatment delays, frustration, and potential health risks.

In Finland, anyone, including friends and family members, can act as an interpreter during medical consultations. One participant preferred to bring their daughter to medical appointments and noted:

“I take my daughter, and she manages in English. She can accompany me up to the doctor’s room. The doctors actually encourage us to bring our children or relatives along when we visit. We don’t usually worry about privacy within the family.” (Participant 19, Woman)

Others placed more trust in informal interpreters from their community rather than professional services:

“I bring an interpreter with me, not an official government interpreter, but a friend I know who can explain my problems to the doctors. If we’re confident that the interpreter has communicated the symptoms, that alone feels like a relief.” (Participant 10, Man)

These narratives highlight the lack of strict regulations in Finland regarding the role of interpreters. Relying on untrained interpreters for comfort may ultimately affect healthcare outcomes, including an increased risk of breaching patient privacy.

While some participants preferred interpreters from their community, others feared breaches of confidentiality in small ethnic groups. Concerns about personal information spreading within the community led some to withhold important health details. One participant shared:

“At first, they provided me with an interpreter, but I wasn’t happy with that because I had to share everything with someone from my own community, and I was worried that information could spread unintentionally. I also felt they didn’t translate things correctly, especially the symptoms, which we had to repeat several times. So, I told the doctors I didn’t want to use an interpreter anymore.” (Participant 23, Woman)

Another participant described how this discomfort prevented open communication:

“It was embarrassing, especially when tulkki (interpreter) was from the same community. I would have preferred to talk to the doctor directly, but I couldn’t because of the language barrier.” (Participant 26, Woman)

However, for some, privacy concerns were secondary to the need for effective communication. One participant explained:

“I don’t fear privacy when I’m sick. I can’t communicate myself with the doctors otherwise. So instead of privacy concerns, the concern was focused on whether or not they could convey my problems.” (Participant 14, Woman)

Although interpreters are essential for facilitating communication, their use often comes with discomfort, miscommunication, and privacy concerns, ultimately affecting the quality of care. Many participants raised concerns like “How can the person who arrived a few years prior to us translate major terms necessary in the medical sector?”

Participants in this study expressed a strong anticipation for Language Concordance (LC) care, thereby avoiding the need for intermediaries such as interpreters. LC refers to situations where healthcare providers and patients communicate directly in a shared native language ​ [40]. They noted that other migrants had found doctors from their ethnic community and believed they received more convenient healthcare treatment.

One participant, who eventually found a Nepali-speaking doctor, expressed his relief:

“After a long search, I finally found a doctor who speaks Nepali and is around my age. After talking to him in Nepali, it felt like half my problems were already solved.” (Participant 10, Man)

In contrast, a woman participant expressed deep dissatisfaction, believing that the loss of her baby was due to the lack of a doctor who spoke a familiar language. She noted:

“I asked several times for a doctor who understood my language so I could explain everything. They kept saying they were looking, but I was always given an interpreter. It was embarrassing to share with them. Only after the tragedy, I saw an Indian doctor. I’m still convinced it wouldn’t have happened if I could have spoken to the doctors in my language.” (Participant 26, Woman)

Despite participants’ expectations of finding healthcare providers from their community, this remains difficult for a small migrant group with limited representation among doctors and nurses.

## Discussion

The results reveal that language barriers, dependence on informal networks, and difficulties with interpreter services collectively influence Nepali migrants’ healthcare experiences in Finland, thereby hindering their ability to access, navigate, and effectively utilise healthcare services.

The study reinforces previous research identifying language barriers as a primary obstacle to healthcare access for migrants [1, 6, 7]. Limited proficiency in Finnish hindered participants from navigating the healthcare system, understanding medical instructions, and effectively communicating with providers, potentially leading to misdiagnoses, medication errors, and delays in care. The delay was also extensive due to misinformation, unfamiliarity with referral pathways, and reliance on informal networks. Furthermore, as evident in previous research [9, 41, 42], the predominance of information in local languages impeded Nepali migrants’ comprehension of the healthcare system.

Many Nepali migrants encountered difficulty understanding the different levels of the healthcare system in Finland, which differs noticeably from the readily available hospital access in Nepal. This lack of understanding of the healthcare system can contribute to perceived discrimination among migrants [7, 18]. For example, some participants interpreted long waiting times and difficulties in accessing specialist care as discriminatory, believing they were deprioritised due to their background​. However, these perceptions may not fully reflect reality. Healthcare access challenges have been worsening across Finland, with the Healthy Finland Survey [43] reporting a 10% increase in access difficulties between 2020 and 2023, even among the general population. This trend is further supported by consistent news reports covering the decline in public healthcare in Finland [44, 45]. Therefore, it is likely that systemic delays affect all residents, not just migrants. Without broader awareness of these structural issues, migrants may misattribute such experiences solely to discrimination.

Although learning the local language is important, some early arrivals did not learn Finnish, either by choice or due to lack of opportunity, leading them to rely on fellow Nepali speakers for communication. In this context, co-ethnic networks played an important role in helping Nepali migrants settle in Finland by providing job opportunities, housing, and daily support, including language assistance and healthcare guidance. While this assistance proved beneficial in the short term [46, 47]​​, many later expressed regret when they encountered difficulties accessing healthcare, experienced workplace exploitation, or faced social exclusion, attributing these challenges to their limited proficiency in Finnish. Media reports [16, 17] highlight cases where Nepali migrant workers experienced poor living conditions and were denied their rights due to employer dependence, illustrating the risks of remaining confined within ethnic enclaves [48]. Similarly, a case involving a suicidal Nepali worker at his workplace [15] suggests the severe mental health challenges faced by migrant workers.

The increasing diversity of Finland’s migrant population has brought into sharper focus the challenges they face in accessing adequate OHC and safe working conditions. In 2023, the Occupational Safety and Health Administration identified several deficiencies in working conditions, with the construction and restaurant sectors, where migrants are heavily employed, recording the highest number of violations [49, 50]. This is further compounded by underutilisation of OHC services ​ [20, 51]​, a disparity that research links to socioeconomic status, with those of higher status having better access [21]. One significant barrier to healthcare access is the misconception that seeking medical attention could negatively impact employment or residency status and poorer long-term health outcomes.

Employees in Finland are legally covered by OHC, and taking sick leave does not impact salary or job security. Likewise, healthcare use does not affect family reunification decisions or residence permit extensions. However, persistent misinformation within migrant networks discourages early healthcare use, contributing to delayed treatment and poorer health outcomes over time. The unique dynamics of Nepali migrants in Finland demonstrate that ethnic networks, although beneficial during early settlement, may hinder long-term integration by limiting access to employment and healthcare rights [15, 52, 53].

As evident in previous research [9, 11], Nepali migrants reported that a scarcity of professional interpreters not only hampered service delivery but, in some cases, postponed urgent procedures. The absence of strict regulations permits anyone to act as ad hoc interpreters, and clients are often encouraged to bring friends or family members for this purpose [9, 25]. While this practice may reduce interpretation accuracy and compromise confidentiality, Nepali migrants did not view the involvement of family members as problematic. This may reflect cultural norms in which sharing personal matters within the family is common. Instead, they were more apprehensive about privacy when interpreters belonged to their own community. Unlike larger groups such as Arabic migrants in Sweden [54] or Somali migrants in Finland [51], who preferred same-sex interpreters from their own communities, Nepali migrants expressed greater unease about confidentiality in such situations.

Moreover, the relatively small size of the Nepali community, where most individuals are acquainted, led clients to question the quality of interpretation services, notably by asking “how someone who had recently arrived in Finland could be proficient in a specialised healthcare language”. These barriers appear less pronounced among larger migrant groups, where clients and interpreters are less likely to know each other.

Nepali migrants, consistent with South Asian and other migrant groups in Nordic countries [51, 54, 55], often prefer LC care, seeking Nepali-speaking providers who understand their cultural background. This preference fosters stronger communication, trust, and cultural understanding, improving health outcomes and removing the need for interpreters [40, 56]. However, due to the small size of the Nepali community in Finland, LC care is largely unavailable, creating an added barrier and contributing to inequalities compared to larger migrant groups with better access to such care.

Although language alone does not ensure optimal healthcare for migrants due to various other barriers, acquiring the local language can significantly improve access to healthcare in destination countries. Beyond facilitating healthcare access, proficiency in the local language empowers migrants and reduces their vulnerability, including to labour exploitation [57], by enhancing their awareness of healthcare rights.

### Strengths and limitations of the study

This study focuses on language barriers and argues that many challenges migrants face are often consequences of inadequate local language skills. This study finds that relying on employers and co-ethnic networks for healthcare information can, in some instances, lead to the denial of healthcare rights. However, whilst some participants experienced exploitation stemming from misinformation about the healthcare system, it is important to avoid broadly emphasising exploitation without supporting evidence. Rather, such instances should be understood as part of a wider array of challenges faced by migrants in destination countries. Therefore, further research on the health and well-being of migrant workers in these sectors is urgently needed to facilitate a more comprehensive understanding of their experiences and to ensure their healthcare needs are adequately addressed.

Acknowledging the study’s small scale, the findings should be interpreted with caution. They may not fully represent the experiences of all Nepali migrants or other minority groups in Finland. Future research, including studies with larger sample sizes and comparative approaches, is needed to strengthen our understanding, allow for more robust generalisations, and inform evidence-based policies. Furthermore, while measures were taken to minimise insider bias, the researcher’s familiarity with participants may have influenced interview data and results. Therefore, this potential impact must be carefully considered when drawing broader conclusions. However, the strength of insider status in qualitative research lies in the ability to build trust, communicate in a shared language, and create a comfortable environment for participants. These factors encouraged greater openness and enabled participants to discuss sensitive topics, such as employment discrimination and challenges related to social integration, with greater ease. Additionally, the use of the purposeful sampling technique, which integrated different sampling methods, facilitated a more targeted participant selection, further enhancing the study’s credibility in a qualitative context.

## Conclusion

This qualitative study explored individual healthcare experiences among Nepali migrants, a minority group in Finland. Despite Finland’s universal healthcare system, disparities in access and utilisation persist among minority migrants, primarily attributable to language barriers, as proficiency significantly affects their ability to navigate the healthcare system.

To foster a more inclusive and effective healthcare system for migrant populations, policymakers should prioritise several key initiatives. First, integration programs must offer comprehensive language courses, ensuring all new arrivals understand their healthcare rights beyond reliance on informal networks. Beyond language acquisition, the availability of professional interpreters is important. Policymakers should establish stringent regulations for interpreter use in healthcare settings to improve accessibility, ensure professionalism, and protect client privacy. Finally, healthcare policies should be tailored at the community level to better address the unique needs of diverse migrant groups, especially those from minority communities. Implementing these measures will promote both integration and equitable access to care, ultimately supporting the well-being of all migrant populations.

## Data Availability

The datasets generated and analysed during the current study are not publicly available, but they are available from the corresponding author upon reasonable request.

## Abbreviations

OHC: Occupational Healthcare
LC: Language Concordance
